# Brain-penetrating peptide and antibody radioligands for proof-of-concept PET imaging of fibrin in Alzheimer’s disease

**DOI:** 10.1186/s41181-025-00383-2

**Published:** 2025-09-08

**Authors:** Dag Sehlin, Ximena Aguilar, Marta Cortés-Canteli, Stina Syvänen, Sara Lopes van den Broek

**Affiliations:** 1 Department of Public Health and Caring Sciences, Uppsala University, Uppsala, 751 85 Sweden; 2 Centro Internacional de Neurociencia Cajal, Consejo Superior de Investigaciones Científicas (CINC, CSIC), Madrid, Spain; 3 Centro Nacional de Investigaciones Cardiovasculares (CNIC), Madrid, Spain

**Keywords:** Fibrin, Alzheimer’s disease, Blood-brain barrier, Vascular, Positron emission tomography

## Abstract

**Background:**

Alzheimer’s disease (AD) is increasingly recognized as a multifactorial disorder with vascular contributions, including a pro-coagulant state marked by fibrin deposition in the brain. Fibrin accumulation may exacerbate cerebral hypoperfusion and neuroinflammation, leading to neurodegeneration. Identifying patients with this pathology could enable targeted anticoagulant therapy. However, current imaging tools lack the specificity and sensitivity to detect fibrin in the brain non-invasively. This study aimed to develop and evaluate brain-penetrating peptide- and antibody-based PET radioligands targeting fibrin to enable individualized treatment strategies in AD.

**Results:**

A fibrin-binding peptide (FBP) was conjugated to the antibody fragment scFv8D3, which targets the transferrin receptor (TfR), to facilitate transcytosis across the blood-brain barrier. FBP-scFv8D3 bound TfR and with modest affinity to fibrin. In vivo studies in Tg-ArcSwe mice, that exhibit fibrin along with brain amyloid-β pathology, and wild-type mice showed that [^125^I]FBP-scFv8D3 retained brain-penetrating properties but did not demonstrate significant fibrin-specific retention. In contrast, the monoclonal antibody 1101 and its bispecific, brain penetrant variant 1101-scFv8D3 exhibited higher fibrin selectivity and TfR binding. Both antibodies showed a trend towards higher brain retention in Tg-ArcSwe mice and [^125^I]1101-scFv8D3 showed a higher brain-to-blood ratio compared to [^124^I]1101. PET imaging with [^124^I]1101 and [^124^I]1101-scFv8D3 revealed low global brain uptake. However, ex vivo autoradiography and regional PET quantification (ROI-to-cerebellum ratios) indicated significant cortical and caudate retention of [^124^I]1101-scFv8D3 in Tg-ArcSwe mice, supporting region-specific target engagement.

**Conclusion:**

This proof-of-concept study demonstrates the feasibility of using bispecific antibody-based PET radioligands to target fibrin in the AD brain. While the FBP-scFv8D3 conjugate showed limited specificity, the bispecific antibody 1101-scFv8D3 exhibited higher brain penetration and fibrin selectivity. These findings support further development of antibody-based imaging tools toward the goal to stratify AD patients who may benefit from anticoagulant therapy.

**Supplementary Information:**

The online version contains supplementary material available at 10.1186/s41181-025-00383-2.

## Introduction

Alzheimer’s disease (AD) neuropathology is characterized by amyloid plaques, tau tangles, and neuroinflammation, which leads to neurodegeneration and cerebral atrophy (Jullienne et al. 2022; Huang and Mucke 2012). In recent years, AD has also been linked to vascular risk factors with accumulating evidence showing a decreased cerebral blood flow in AD patients. More specifically, the level of cerebral hypoperfusion in the AD brain has been correlated to the degree of dementia. This may be partially due to a significant pro-coagulant state present in AD, driven by the formation and deposition of fibrin, the main protein of blood clots (Farkas and Luiten 2001; Johnson et al. 2005; Staffen et al. 2009). Fibrin is found both in the vasculature, where it builds up from the blood, and inside the brain, in the parenchyma. The extent of fibrin pathology varies greatly among individuals with AD, likely reflecting the heterogeneity of the patient population. The pro-coagulant state is increasingly recognized as a factor in AD progression, and anticoagulant therapies aimed at reducing fibrin(ogen) levels may offer a promising strategy to normalize cerebral blood flow (Cortes-Canteli et al. 2019). However, since this pro-coagulant state is only present in a subset of patients, individualized treatment approaches are essential. Taking into consideration that anticoagulants are already widely available, their repurposing for AD treatment is both feasible and cost-effective, particularly in comparison to newly approved AD immunotherapies (Toribio-Fernandez et al. 2024; Grossmann 2021). To identify the patients that can benefit from anticoagulant treatment, novel sensitive and specific diagnostic tools must be developed (Cortes-Canteli et al. 2015). 

Positron emission tomography (PET) is a non-invasive nuclear molecular imaging technique that enables sensitive quantification and visualization of disease targets (Basu et al. 2011; Piel et al. 2014; Ametamey et al. 2008; Hicks et al. 2006; Kristensen and Herth 2017). Currently, most PET radioligands are small molecules. To cross the blood-brain barrier (BBB), radioligands must be sufficiently lipophilic, which can result in off-target distribution within brain tissue. Moreover, small molecule radioligands often exhibit limited target specificity and sensitivity, reducing the reliability and accuracy of PET imaging.

Peptide- and antibody-based PET ligands offer a promising alternative due to their high target specificity, selectivity, and low non-displaceable binding (Sehlin et al. 2016, 2017; He et al. 2020; Psimadas et al. 2018). However, their relatively large size prevents them from efficiently crossing the BBB. This limitation underscores the need for innovative strategies to enhance brain uptake. One of the most widely explored approaches is receptor-mediated transcytosis, which employs bispecific constructs that bind both the intrabrain target molecule and a receptor expressed on the BBB, such as the transferrin receptor (TfR), which facilitates transcytosis of the ligand across the BBB and into the brain (Sehlin et al. 2019, 2025; Bonvicini et al. 2022; Faresjö et al. 2021). 

In this proof-of-concept study, we aim to develop brain-penetrating fibrin-targeting PET radioligands capable of detecting the pro-coagulant state in AD. More specifically, we developed a bispecific fibrin-binding peptide as well as a bispecific antibody ligand and evaluated their potential to identify fibrin in the brain, with a future aim to individualize treatment for AD patients that can benefit from anticoagulant therapy.

## Results

This study investigated two approaches for developing PET radioligands to image fibrin in the living brain: one based on a fibrin-binding peptide (FBP) and the other on a fibrin-binding antibody (1101). Both FBP and 1101 were conjugated to the mouse transferrin receptor (mTfR)-binding Fab fragment 8D3 for facilitated brain delivery.

### Conjugation of FBP to scFv8D3

A fibrin binding peptide (FBP), previously used to detect fibrin in an experimental model of mural thrombosis was synthesized with an NHS-ester at the peptide N-terminus (Figure S1) (Blasi et al. 2015; Oliveira et al. 2015). The NHS-ester was used as a handle for conjugation to lysine residues on scFv8D3. This method has been widely used as a bioconjugation strategy and allows the attachment of multiple probes to one antibody or antibody fragment (Lopes et al. 2022; Cardinale et al. 2019). This method’s lack of selectivity imposes a risk of interference with the binding properties of the antibody (fragment) if conjugations are made within the binding region (CDR region) (Cook et al. 2016). Therefore, scFv8D3 was designed with a lysine-rich FlagTag the C-terminus to steer the site of modification away from the CDR (Meier et al. 2021). The conjugation efficiency was tested using different FBP-NHS ester: scFv8D3 ratios, using 15–50 equivalents (eq.) FBP-NHS excess.

### Binding of FBP-scFv8D3 to fibrin and TfR

**Fig. 1:**
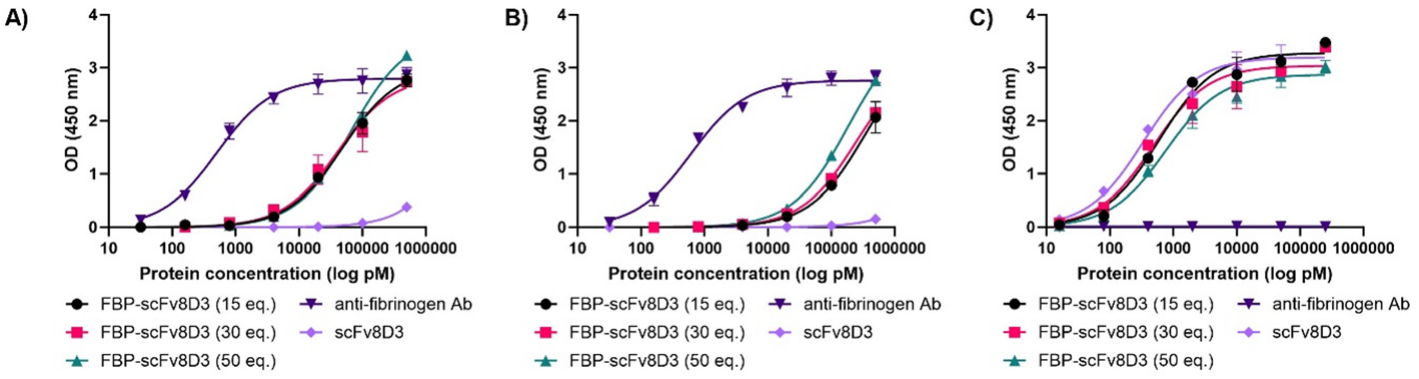
Indirect ELISA results showing FBP-scFv8D3 binding towards **A**) fibrin, **B**) fibrinogen and **C**) mTfR. The anti-fibrinogen antibody showed much higher binding towards both fibrin and fibrinogen compared to the FBP-scFv8D3 constructs and no binding towards mTfR, whereas scFv8D3 bound only to mTfR and not to fibrin or fibrinogen

Enzyme-linked immunosorbent assay (ELISA) was performed to confirm conjugation of FBP to scFv8D3 and assess the potential impact on the affinity of three FBP-scFv8D3 conjugates (15, 30 and 50 eq.) towards fibrin, fibrinogen and mTfR. Commercially available anti-fibrinogen antibody and scFv8D3 were used as controls. The anti-fibrinogen antibody showed equal affinity towards fibrin and fibrinogen, with no binding towards mTfR, whereas scFv8D3 bound to mTfR but not to fibrin or fibrinogen (Fig. 1). All three FBP-scFv8D3 constructs bound fibrin, however, with substantially lower affinity compared to the control antibody. The FBP-scFv8D3 constructs exhibited binding to mTfR with affinities comparable to that of unmodified scFv8D3, indicating that FBP was successfully conjugated without compromising mTfR binding. FBP-scFv8D3 (50 eq.) showed approximately 20–30% stronger fibrin binding compared to the lower-loaded constructs but exhibited an approximate 120% reduction in mTfR binding affinity relative to unmodified scFv8D3. The 15 eq. and 30 eq. versions showed ~ 75% and ~ 50% reductions in mTfR binding affinity, respectively. This can be explained by the addition of more FBPs to the scFv8D3 moiety, which might increase the fibrin binding but can also interfere with the scFv8D3 binding to mTfR. Notably, all FBP-scFv8D3 also showed some degree of binding to fibrinogen, which implies that conjugation to scFv8D3 may reduce the peptide’s affinity to fibrin and thereby the selectivity towards fibrin over fibrinogen.

### Biodistribution of FBP-scFv8D3

**Fig. 2:**
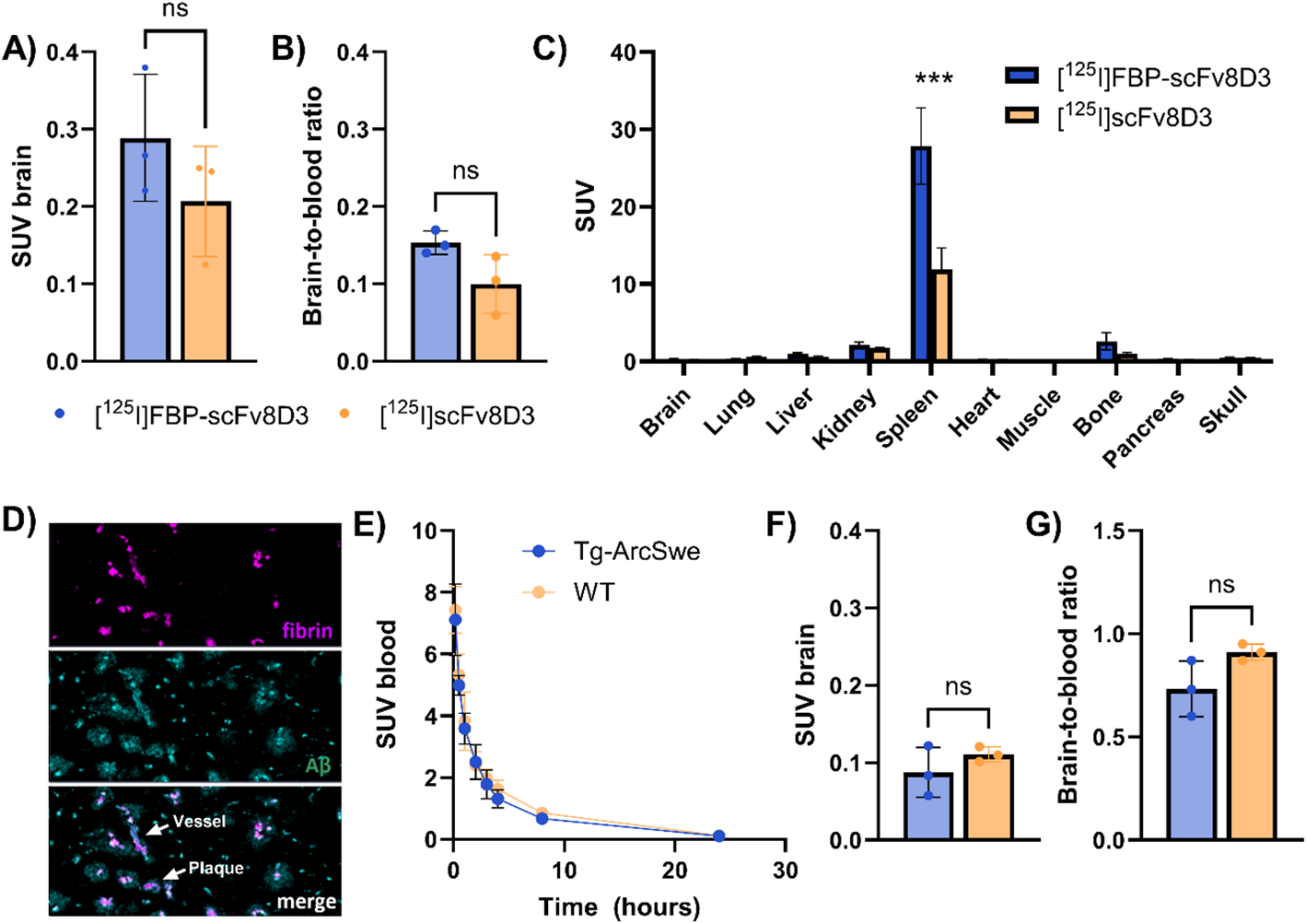
Ex vivo evaluation of [^125^I]FBP-scFv8D3. **A**) Brain uptake, expressed as standardized uptake value (SUV), of [^125^I]FBP-scFv8D3 and [^125^I]scFv8D3 in WT mice. **B**) Brain-to-blood ratio in WT mice 2 h after injection of [^125^I]FBP-scFv8D3 and [^125^I]scFv8D3. **C**) [^125^I]FBP-scFv8D3 and [^125^I]scFv8D3 biodistribution to peripheral organs at 2 h after injection in WT mice. **D**) Immunofluorescent staining of fibrin (magenta; antibody 1101) in combination with Aβ staining (cyan; LCO HS-84), confirming the presence of fibrin in Aβ plaques and brain vessels. **E**) Blood concentrations of [^125^I]FBP-scFv8D3 in Tg-ArcSwe and WT mice over 24 h. **F**) Brain uptake of [^125^I]FBP-scFv8D3 at 24 h after injection in Tg-ArcSwe and WT mice. **G**) Brain-to-blood ratio of [^125^I]FBP-scFv8D3 at 24 h after injection Tg-ArcSwe and WT mice

Brain uptake and biodistribution studies were performed with FBP-scFv8D3, conjugated at 25 eq. peptide excess. This conjugation ratio was selected as a compromise between enhanced fibrin binding at higher peptide loads and the progressive reduction in mTfR binding observed with increasing peptide conjugation. After iodine-125 (^125^I)-radiolabeling of FBP-scFv8D3, brain and blood concentrations were first evaluated in WT mice at 2 h after injection, using the non-conjugated scFv8D3 as control. Ex vivo analyses in WT animals showed similar brain uptake and brain-to-blood ratio for [^125^I]FBP-scFv8D3 and [^125^I]scFv8D3 at 2 h after injection, indicating that the addition of FBP moieties to the scFv8D3 did not significantly interfere with the brain penetrating capacities of scFv8D3 (Fig. 2A-B). Notably, the 2 h brain uptake and brain-to-blood ratio of [^125^I]FBP-scFv8D3 were approximately 10- to 30-fold higher than those previously observed for the FBP peptide alone, confirming the improved brain delivery conferred by the scFv8D3 shuttle (Ciesienski et al. 2013). Analysis of biodistribution to peripheral organs indicated high concentrations of both ligands in spleen, consistent with previous studies on TfR binding ligands (Fig. 2C). Next, [^125^I]FBP-scFv8D3 was evaluated in the Tg-ArcSwe mouse model of Aβ pathology, which has fibrin deposits associated both with parenchymal plaques and the brain vasculature (Fig. 2D). Measurements of [^125^I]FBP-scFv8D3 blood concentrations over 24 h showed no difference between Tg-ArcSwe and WT mice (Fig. 2E). Brain concentrations of [^125^I]FBP-scFv8D3 measured 24 h post injection were overall low, with no difference between Tg-ArcSwe and WT mice (Fig. 2F). Similarly, no difference was seen in the brain-to-blood ratio (Fig. 2G), implying no significant binding to fibrin clots in the brain of Tg-ArcSwe mice.

### Binding of 1101 and 1101-scFv8D3 to fibrin and TfR

**Fig. 3:**
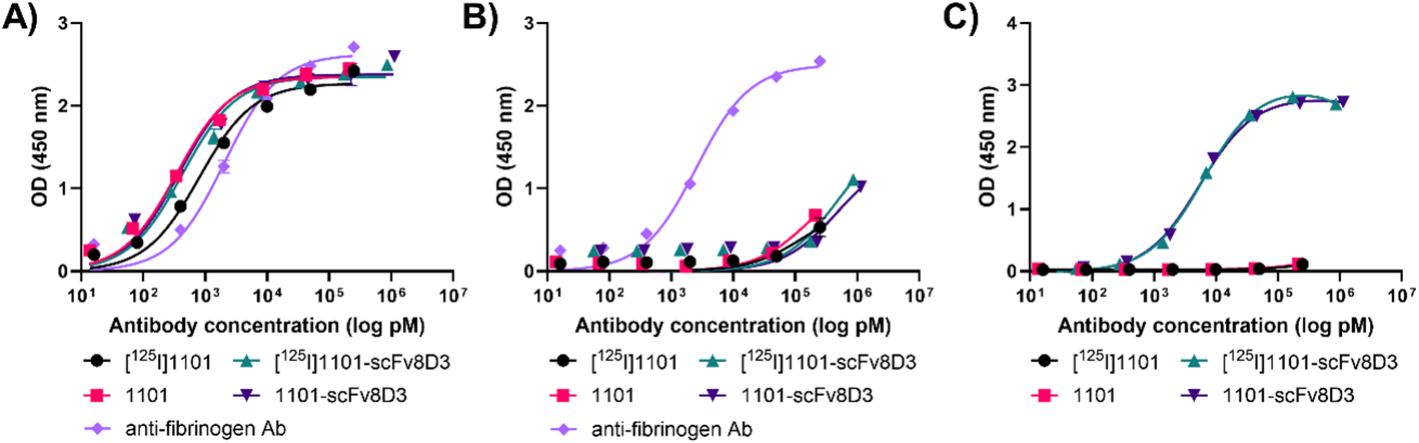
In vitro evaluation of 1101 and 1101-scFv8D3. 1101 and 1101-scFv8D3 were evaluated, before and after radiolabeling, with indirect ELISA for their binding towards **A**) fibrin; **B**) fibrinogen; and **C**) mTfR. A commercially available anti-fibrinogen antibody was used as control in **A** and **B**

While a relatively small peptide such as FBP may be greatly affected by conjugation to scFv8D3, antibodies generally tolerate such fusions. Thus, the monoclonal fibrin-specific antibody 1101 was evaluated along with its bispecific variant 1101-scFv8D3, where scFv8D3 was attached to each of the light chains of antibody 1101, to enhance its brain uptake (Hultqvist et al. 2017). ELISA assays showed highly selective binding of antibody 1101 and 1101-scFv8D3 towards fibrin over fibrinogen, with similar affinity as the anti-fibrinogen antibody control (Fig. 3A-B). 1101-scFv8D3 also bound to mTfR, while 1101 did not (Fig. 3C).

### Biodistribution of 1101 and 1101-scFv8D3

**Fig. 4:**
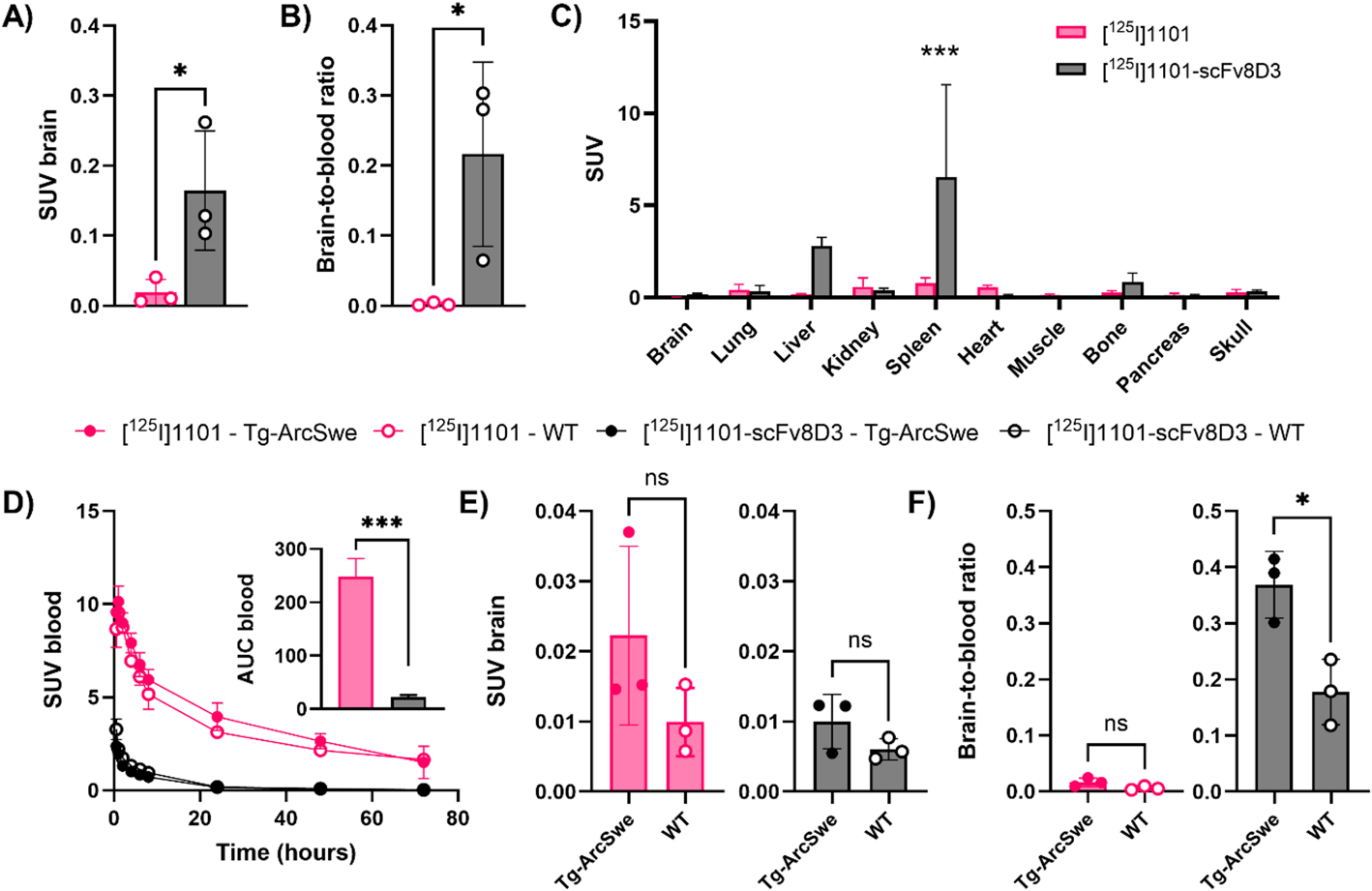
Ex vivo evaluation of [^125^I]1101 and [^125^I]1101-scFv8D3. **A**) Brain uptake in WT mice 2 h after injection of [^125^I]1101 and [^125^I]1101-scFv8D3. **B**) Blood-to-brain ratio in WT mice after 2 h after injection of [^125^I]1101 and [^125^I]1101-scFv8D3. **C**) Biodistribution of [^125^I]1101 and [^125^I]1101-scFv8D3 in WT mice at 2 h after injection. **D**) Blood concentration of [^125^I]1101 and [^125^I]1101-scFv8D3 in Tg-ArcSwe and WT mice over 72 h. Inset: Area under the curve for [^125^I]1101 and [^125^I]1101-scFv8D3 blood concentration between 0 and 72 h. **E**) Brain concentrations and **F**) brain-to-blood ratio of [^125^I]1101 and [^125^I]1101-scFv8D3, 72 h after injection in Tg-ArcSwe and WT mice

Antibodies 1101 and 1101-scFv8D3 were radiolabeled with iodine-125 and injected in WT mice. Both the brain uptake (Fig. 4A) and the brain-to-blood ratio (Fig. 4B) were higher for the bispecific 1101-scFv8D3 than for 1101, indicating better brain-penetrating properties. Biodistribution to peripheral organs (Fig. 4C) showed higher uptake in the spleen of the bispecific antibody, again indicating TfR binding in the spleen. Next, [^125^I]1101 and [^125^I]1101-scFv8D3 were evaluated in the Tg-ArcSwe mouse model of Aβ pathology, which has fibrin deposits associated both with parenchymal plaques and the brain the vasculature (Fig. 2D), in comparison to WT mice that have less fibrin deposits in the brain (Figure S2). In line with previous observations for TfR-binding bispecific antibodies, [^125^I]1101-scFv8D3 showed a rapid clearance from blood, significantly reducing its systemic exposure compared with [^125^I]1101 (Fig. 4D). At 72 h post administration, similar brain retention was observed for both antibodies, with no significant differences between Tg-ArcSwe and WT mice, although both antibodies showed a trend towards higher retention in Tg-ArcSwe mice (Fig. 4E). Due to its fast blood clearance, [^125^I]1101-scFv8D3 displayed a significantly higher brain-to-blood ratio compared with [^125^I]1101, and also a difference between Tg-ArcSwe and WT mice (Fig. 4F). The brain-to-blood ratio is especially relevant for PET imaging, where high imaging contrast is dependent on the specific binding to the target (brain) vs. low background (blood).

### PET imaging with 1101 and 1101-scFv8D3

**Fig. 5:**
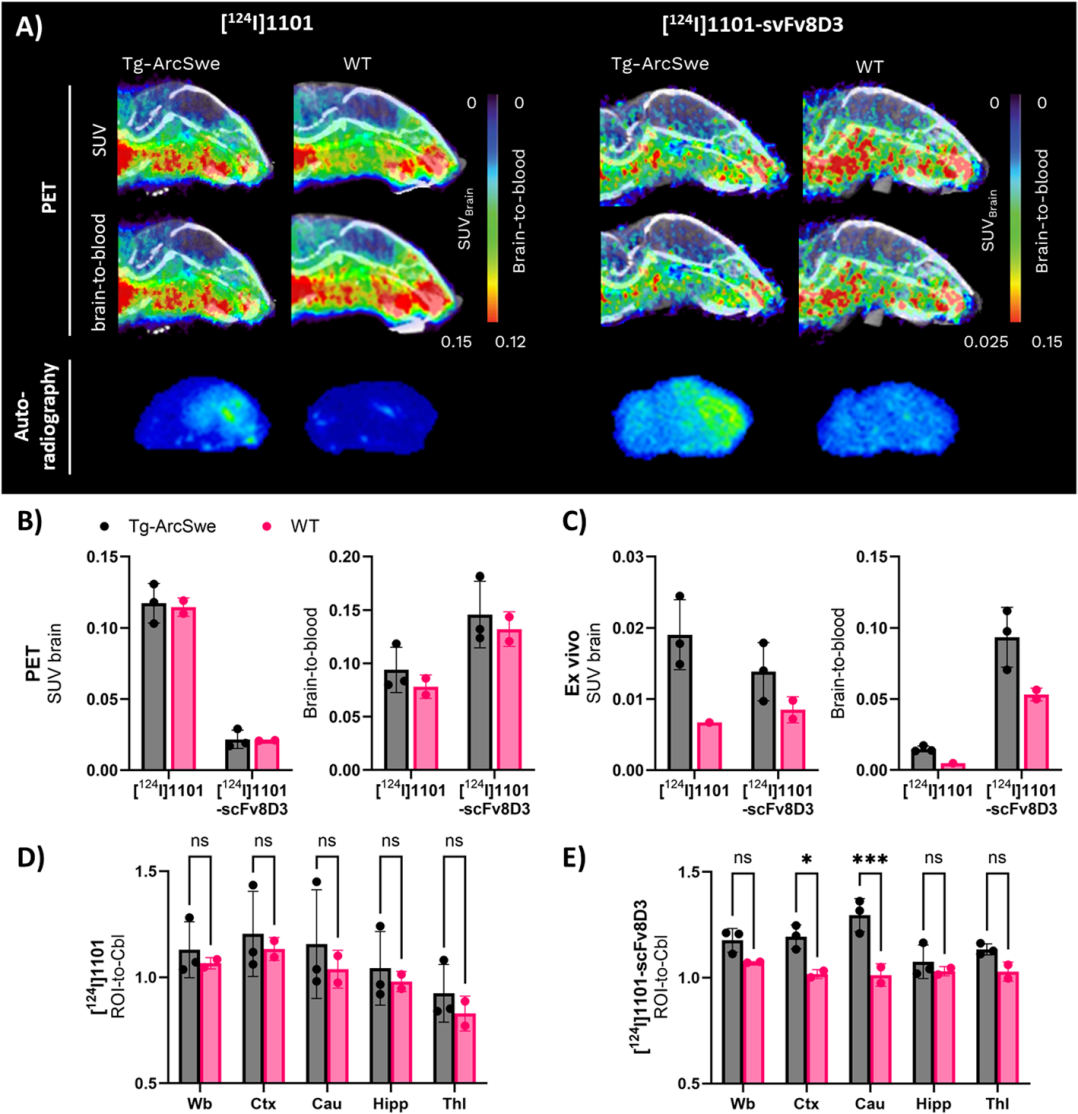
ImmunoPET with [^124^I]1101 and [^124^I]1101-scFv8D3. **A**) Representative sagittal PET images of Tg-ArcSwe and WT mice acquired 72 h post injection of [^124^I]1101 or [^124^I]1101-scFv8D3, expressed as standardized uptake value (SUV, upper panel) or brain to blood ratio (middle panel). Note the different scales for the two antibody ligands. Ex vivo autoradiography (lower panel) from saline perfused, PET scanned mice. **B**) Quantification of PET images from **A**), expressed as SUV and brain-to-blood concentration ratio. **C**) Ex vivo quantification of [^124^I]1101 and [^124^I]1101-scFv8D3 concentration in brain tissue from PET scanned mice in **A**), expressed as SUV and brain-to-blood concentration ratio. Quantification of brain regions in relation to cerebellum (Cbl), expressed as ROI-to-Cbl ratios, in the whole brain (Wb), cortex (Ctx), caudate (Cau), hippocampus (Hipp) and thalamus (Thl), for (**D**) [^124^I]1101 and (**E**) [^124^I]1101-scFv8D3

Antibodies 1101 and 1101-scFv8D3 were radiolabeled with iodine-124 (^124^I) and administered to Tg-ArcSwe and WT mice for immunoPET imaging. PET images acquired 72 h post administration revealed overall low brain concentrations of both antibodies, relative to surrounding tissues, with no clear visual differences between Tg-ArcSwe and WT mice. Ex vivo autoradiography images, however, suggested a specific retention of both antibodies in the brains of Tg-ArcSwe mice, most notably in the cortex (Fig. 5, Figure S3). Quantification of PET data confirmed the visual impression from PET images, with equal average brain concentrations in Tg-ArcSwe and WT mice (Fig. 5B). In line with the autoradiography, ex vivo quantification of radioactivity in post mortem tissue revealed elevated concentrations and brain-to-blood ratios of both antibodies in Tg-ArcSwe mice compared to WT (Fig. 5C). The Tg-ArcSwe model is largely spared from development of AD related pathology in the cerebellum, making it suitable as a reference region (Sehlin et al. 2016). Quantification of regional PET signals using the cerebellum (Cbl) as a reference region (ROI-to-Cbl ratios) revealed significantly higher uptake of [¹²⁴I]1101-scFv8D3 in the cortex (Ctx) and caudate (Cau) regions of Tg-ArcSwe mice compared to WT controls. No such significant differences were observed for the monospecific [¹²⁴I]1101 (Fig. 5D-E). These regional findings further support target engagement by 1101-scFv8D3 in areas relevant to AD pathology.

## Discussion

This study presents a significant advancement in the development of molecular imaging tools for Alzheimer’s disease (AD), particularly targeting the vascular component of the disease through fibrin imaging. The rationale stems from increasing evidence that a subset of AD patients exhibit a pro-coagulant state characterized by fibrin deposition, which contributes to neurovascular dysfunction, impaired cerebral perfusion, neuroinflammation and potentially accelerates neurodegeneration. Detecting this pathology non-invasively could enable stratification of patients for anticoagulant therapy, a promising and cost-effective intervention given the widespread availability of such drugs. In addition, it could be used to identify patients that may benefit from anti-fibrin immunotherapy (Ryu et al. 2018). 

The peptide-based radioligand FBP-scFv8D3 was designed to combine fibrin specificity with brain-penetrating capabilities via TfR-mediated transcytosis. While the construct retained its ability to cross the BBB via TfR-mediated transcytosis, as evidenced by comparable brain uptake to the parent scFv8D3, its binding affinity to fibrin was modest and lacked sufficient selectivity over fibrinogen. This limitation is critical, as high off-target binding in the periphery could compromise imaging specificity. Moreover, in vivo studies did not show significant fibrin-specific retention in Tg-ArcSwe mice. Taken together, the affinity and selectivity of the FBP-scFv8D3 conjugate was insufficient for effective in vivo imaging.

In contrast, the monoclonal antibody 1101 and its bispecific variant 1101-scFv8D3 demonstrated high in vitro affinity and selective binding for fibrin over fibrinogen. The bispecific format enabled BBB transcytosis, yielding increased brain uptake compared to the monospecific 1101 antibody, without compromising target binding. The brain uptake at 2 h post-injection was somewhat lower for 1101-scFv8D3 compared to the FBP-scFv8D3 peptide conjugate, which could reflect the larger size of the antibody and correspondingly slower kinetics. Notably, initial biodistribution studies showed a trend for both antibodies towards higher brain retention in Tg-ArcSwe over WT mice, indicating potential for fibrin specific binding. The bispecific [^125^I]1101-scFv8D3 also exhibited an elevated brain-to-blood ratio, which was significantly higher in Tg-ArcSwe compared to WT mice. Although PET imaging showed low absolute brain uptake by visual inspection, ex vivo autoradiography revealed retention in transgenic mice, particularly in the cortex, a region commonly affected in this model, supporting the hypothesis of target engagement. This observation was further corroborated by regional PET quantification, where ROI-to-Cbl ratios demonstrated statistically significant differences between genotypes in the cortex and caudate regions for [^124^I]1101-scFv8D3. This finding underscores the potential specific detection of fibrin accumulation by the bispecific antibody in specific brain regions, even in the context of low overall brain uptake. In contrast, the monospecific 1101 antibody did not reveal any regional differences, highlighting the enhanced sensitivity provided by the bispecific format. The brain uptake of [^124^I]1101 and [^124^I]1101-scFv8D3 at 2 h post-injection was comparable to that observed in our previous studies comparing conventional and bispecific antibodies that target amyloid beta (Aβ), and suggests efficient initial brain delivery of [^124^I]1101-scFv8D3 (Hultqvist et al. 2017). The low global brain retention of [^124^I]1101-scFv8D3 observed at 3 days is therefore likely attributable to the lower abundance of accessible fibrin pathology in Tg-ArcSwe mice compared to the strongly pronounced Aβ deposition.

These findings stress the importance of molecular format in radioligand design. While peptides offer advantages in terms of size and synthetic flexibility, their binding characteristics may be more susceptible to modification upon conjugation. Antibodies, particularly when engineered as bispecific constructs, provide a more robust platform for achieving both target specificity and brain delivery. The use of TfR-mediated transcytosis is particularly promising, as it allows for the delivery of large biologics across the BBB, a major hurdle in CNS drug and imaging agent development. It should be noted, however, that fibrin is likely present on both the luminal and abluminal sides of the BBB. Therefore, a conventional IgG without TfR-mediated transcytosis could still be sufficient, which may explain the relatively small differences observed in brain retention between [¹²⁵I]1101 and [¹²⁵I]1101-scFv8D3. Nonetheless, the faster systemic clearance of the bispecific variant, [¹²⁵I]1101-scFv8D3, is advantageous from a PET imaging perspective, as it contributes to more rapid background clearance and improved contrast between specific and non-specific signals. However, several limitations must be addressed in future studies. First, the sample sizes in animal experiments were relatively small, limiting statistical power. Increasing the number of animals and including additional time points could provide more definitive evidence of target engagement and pharmacokinetics. Here, it was not possible to increase the number of included animals since the antibody proved to be very difficult to express. Thus, other expression systems, or other antibody formats, should be investigated for obtaining larger quantities of the antibodies. Second, the radiolabeling strategy and choice of isotope may influence imaging sensitivity and resolution. Optimization of radiochemistry, including site-specific labeling and use of isotopes with more favorable decay properties, could enhance imaging performance.

Additionally, while the Tg-ArcSwe model recapitulates key aspects of AD pathology, including variable degrees of fibrin deposition, it remains primarily a model of Aβ pathology, and thus a simplified representation of the human disease. Validation in other models and, ultimately, in human tissue or clinical studies will be essential to confirm the translational potential of this radioligand.

## Conclusion

In conclusion, this study provides a compelling proof-of-concept for the use of bispecific antibody-based PET radioligands to detect fibrin in the AD brain. The results support further development and optimization of such tools for patient stratification and monitoring of anticoagulant therapy. Notably, regional PET quantification, expressed as ROI-to-Cbl ratios, revealed significant retention of the bispecific antibody 1101-scFv8D3 in cortex and caudate regions of Tg-ArcSwe mice, providing evidence of localized fibrin detection. By enabling personalized treatment strategies, this approach could significantly improve outcomes for a subset of AD patients and represents a promising direction in the evolving landscape of precision medicine in neurodegenerative diseases.

## Materials and methods

### Conjugation of FBP to scFv8D3

Single chain fragment variable scFv8D3-FlagTag was produced according to previously published procedures (Meier et al. 2021). FBP functionalized with an NHS ester at the C-terminal was produced on special request by ThermoFischer. FBP was stored in DMSO and freshly diluted in PBS before the conjugation reaction. Sodium carbonate buffer (1 M, pH 8.0) was added to an Eppendorf tube containing scFv8D3-FlagTag (1-1.20 mg/mL in PBS, 20–100 uL) to give a final concentration of 30 mM. FBP dissolved in PBS containing. 10% DMSO was added to the mixture using 5-100 equiv. peptide-scFv8D3 ratio. The mixture was incubated at room temperature for 2 h at 600 RMP. The solution was purified to remove unreacted FBP using Zeba spin desalting columns (7 K MWCO, 0.5 mL, 89882, Thermofisher) and eluted in PBS (pH 7.4). Final protein concentration was measured with spectrophotometry (DeNovix spectrophotometer DS-11).

### Expression of antibodies 1101 and 1101-scFv8D3

Antibody 1101 was designed as a mouse IgG2c backbone with the variable domains from a previously described fibrin specific antibody (Matsumura et al. 2020). Its bispecific variant 1101-scFv8D3 was designed with scFv8D3 fused to the C-terminal ends of the 1101 light chains. Both antibodies were expressed in Expi293 cells and purified using protein A chromatography, according to a previously described protocol (Hultqvist et al. 2017; Dahlén et al. 2025). 

### SDS-PAGE

SDS-PAGE analyses were conducted to confirm the size and integrity of the antibodies and FBP-scFv8D3 conjugates. The protein samples were mixed with Bolt LDS sample buffer (ThemoFischer, 13276499), heated for 2 min at 95 °C, and loaded onto 4–12% Bolt Bis-Tris Plus Gels (ThermoFischer) and run for 22 min at 200 V in MES buffer (ThermoFischer, 13266499). PageRuler™ Plus Prestained Protein was added as a molecular weight standard (ThermoFischer, 11832124). Afterwards, the gel was removed and washed in dH2O and stained with InstantBlue (Abcam, ab119211). Images of the gels were taken with a ChemiDoc XRS + Gel Imaging System (BioRad) and analyzed with ImageLab (BioRad).

### ELISA

**Fibrin.** Fibrin ELISA assay methodology was adapted from Hanaoka et al. (Hanaoka et al. 2021) The 96-well half-area plates (Corning Inc., New York, NY) were coated overnight at 4°C with fibrinogen (50 µL/well, 10 µg/mL diluted in PBS, Sigma-Aldrich F3879). A mixture of 0.05 U/mL thrombin (Sigma-Aldrich 605195), 7mM L-cysteine and 1 mM CaCl_2_ with a total volume of 50 µL/mL was added and the plates were incubated for 2 h at 37°C. **Fibrinogen.** The 96-well half-area plates (Corning Inc., New York, NY) were coated overnight at 4°C with fibrinogen (50 µL/well, 10 µg/mL diluted in PBS, Sigma-Aldrich F3879). **Mouse transferrin receptor.** The 96-well half-area plates (Corning Inc., New York, NY) were coated overnight at 4°C with mTfR (50 µL/well, 5 µg/mL diluted in PBS). **Blocking.** The wells were blocked with 1% BSA in PBS for 1 h. The proteins were diluted in incubation buffer (PBS containing 0.1% BSA, 0.05% Tween-20 and 0.15% Kathon) **Primary antibody.** The plates were washed with wash buffer (phosphate buffer with 0.1% Tween-20 and 0.15% Kathon, pH 7.5). 1101, 1101-scFv8D3, FBP-scFv8D3, their radiolabeled equivalent, and control antibodies were serial diluted in incubation buffer (PBS with 0.1% BSA, 0.05% Tween-20 and 0.15% Kathon) from 250 nM (for fibrin and fibrinogen coated plates) or 50 nM (for mTfR and mIgG coated plates) to 3.2 pM and incubated for 2 hours. **Secondary antibody**. The plates were washed with wash buffer and detected with horseradish peroxidase (HRP)-coupled goat anti-mouse IgG-F(ab’)2 (1:2000, Jackson ImmunoResearch Laboratories, West Grove, PA, #115-035-006) or rabbit anti-goat IgG (1:4000, Thermofischer, #31402). **Plate development**. Signals were developed with K blue aqueous TMB substrate (Neogen Corp., Lexington, KY), quenched with 1M H_2_SO_4_ and read with a spectrophotometer at 450 nm. The data was analyzed using GraphPad Prism.

### Animals

The Tg-ArcSwe mouse model harbors the Arctic (E693G) and Swedish (KM670/671NL) APP mutations and is maintained on a C57BL/6 background. Tg-ArcSwe mice show elevated levels of soluble Aβ protofibrils at a young age and abundant and rapidly developing plaque pathology starting at around 6 months of age (Philipson et al. 2009; Lord et al. 2006). Both males and females were used and WT littermates were used as control animals. The animals were housed in rooms with controlled temperature and humidity in an approved facility at Uppsala University with *ad libitum* access to food and water. All procedures described in this paper were approved by the Uppsala County Animal Ethics board (5.8.18–20401/2020), following the rules and regulations of the Swedish Animal Welfare Agency and in compliance with the European Communities Council Directive of 22 September 2010 (2010/63/EU).

### Radiochemistry

FBP-scFv8D3 and the two antibodies 1101 and 1101-scFv8D3 were radiolabeled by direct radioiodination of iodine-125 using the Chloramine-T method (Greenwood et al. 1963). FBP-scFv8D3 (70 µg, 58.3 µL, 2500 pmol), 1101 (70 µg, 25.5 µL, 467 pmol) or 1101-scFv8D3 (100 µg, 102 µL, 476 pmol) was mixed with 13.7–15.4 MBq [^125^I]NaI (Perkin-Elmer Inc Waltham, MA, USA) and 10 µg Chloramine-T (Sigma Aldrich, Stockholm, Sweden) and the reaction mixture was diluted in PBS to a final volume of 260 µL. The reaction was incubated for 90 s at room temperature and subsequently quenched with 20 µg sodium metabisulfite (Sigma Aldrich, Stockholm, Sweden). The product was diluted with PBS to 500 µL and unreacted iodine, Chloramine-T and sodium metabisulfite were removed with a NAP-5 size exclusion column (GE Healthcaer, Uppsala, Sweden) with a total elution volume of 1 mL PBS. The ^125^I-iodination resulted in molar activities of 5.3 MBq/nmol, 24.4 MBq/nmol, and 13.7 MBq/nmol for FBP-scFv8D3, 1101, and 1101-scFv8D3, respectively.

For PET imaging, 1101 and 1101-scFv8D3 were labeled with iodine-124. 120 µl [^124^I]NaI stock solution was preincubated 15 min with 12 µl cold NaI (100 µM), then neutralized with 24 µl of 0.5% acetic acid and 17 µl 10xPBS before addition of 145 µg of 1101 or 160 µg of 1101-scFv8D3. Each reaction was started by the addition of Chloramine T to a final concentration of 75 µg/ml and stopped after 2 min with Na-metabisulphite at 150 µg/ml. Labeled antibodies were purified with NAP-5 columns as above. The ^124^I-iodination resulted in molar activities of 36.4 MBq/nmol for 1101 and 44.1 MBq/nmol for 1101-scFv8D3. All labeled compounds showed a radiochemical purity > 95%. Radiochemical purity was assessed using radio-TLC on iTLC-SG paper (Agilent, SGI0001) with 70% acetone in water as the mobile phase. TLC plates were exposed to phosphor imaging screens and analyzed using a Cyclone Storage Phosphor System (Packard Instruments Co., Meriden, CT, USA). Quantification was performed with OptiQuant software (version 3.00, Packard Instruments Co.), using the percentage of total lane signal to determine peak intensities. The retention factors (Rf) were: [^124/125^I]1101/[^124/125^I]1101-scFv8D3: Rf = 1, Free [^124/125^I]NaI: Rf = 1. *Biodistribution*.

Mice were injected intravenously (i.v.) in the tail vein with either 1.0 MBq ± 0.15 [^125^I]FBPscFv8D3; 0.42 MBq ± 0.036 [^125^I]scFv8D3; 1.0 MBq ± 0.18 [^125^I]1101; or 0.56 MBq ± 0.11 [^125^I]1101-scFv8D3. One cohort of WT mice (3–24 months, *n* = 3 per group) were sacrificed 2 h post-injection to assess general brain uptake independent of pathology. A second cohort of aged Tg-ArcSwe mice and WT littermates was used to asses biodistribution and brain target engagement 24–72 h after injection (Tg-ArcSwe: 18–24 months, *n* = 9; WT: 18–24 months, *n* = 9). Blood samples (8 µL) were obtained from the tail vein of selected individuals at 0.5, 1, 2, 5 4, 6, 8, 24, 48 h to investigate the radioactivity concentration in blood over time. After 2, 24–72 h, mice were anaesthetized with isoflurane and a terminal blood sample was taken from the heart, followed by transcardial perfusion with 40 mL of 0.9% NaCl for 2.5 to clear the brain and organs from blood. Thereafter, lung, liver, kidney, whole heart, pancreas, spleen, femoral muscle, femoral bone, skull bone and submandibular glands were isolated to evaluate the biodistribution of the radiolabeled compounds. The brain was divided into left and right hemispheres, and the left hemisphere was further divided into cerebrum and cerebellum. The brain samples were immediately frozen on dry ice. The radioactivity of the samples was measured with a gamma-counter (2480 Wizard™, Wallac Oy PerkinElmer, Turku, Finland). Protein concentrations were expressed as percent of injected dose per gram tissue (%ID/g) corrected for body weight, referred to as standardized uptake value (SUV).

1 $$\:SUV=\frac{\text{r}\text{a}\text{d}\text{i}\text{o}\text{a}\text{c}\text{t}\text{i}\text{v}\text{i}\text{t}\text{y}\:\text{p}\text{e}\text{r}\text{g}\text{r}\text{a}\text{m}\:\text{t}\text{i}\text{s}\text{s}\text{u}\text{e}}{\text{i}\text{n}\text{j}\text{e}\text{c}\text{t}\text{e}\text{d}\:\text{r}\text{a}\text{d}\text{i}\text{o}\text{a}\text{c}\text{t}\text{i}\text{v}\text{i}\text{t}\text{y}\:\text{p}\text{e}\text{r}\:\text{g}\text{r}\text{a}\text{m}\:\text{b}\text{o}\text{d}\text{y}\:\text{w}\text{e}\text{i}\text{g}\text{h}\text{t}\:}$$

The brain-to-blood ratio was calculated by dividing the radioactivity in the brain per gram brain and the radioactivity in the blood per gram blood.

2 $$\:\text{B}\text{r}\text{a}\text{i}\text{n}-\text{t}\text{o}-\text{b}\text{l}\text{o}\text{o}\text{d}\:\text{r}\text{a}\text{t}\text{i}\text{o}=\frac{\text{r}\text{a}\text{d}\text{i}\text{o}\text{a}\text{c}\text{t}\text{i}\text{v}\text{i}\text{t}\text{y}\:\text{p}\text{e}\text{r}\:\text{g}\text{r}\text{a}\text{m}\:\text{b}\text{r}\text{a}\text{i}\text{n}}{\text{r}\text{a}\text{d}\text{i}\text{o}\text{a}\text{c}\text{t}\text{i}\text{v}\text{i}\text{t}\text{y}\:\text{p}\text{e}\text{r}\:\text{g}\text{r}\text{a}\text{m}\:\text{b}\text{l}\text{o}\text{o}\text{d}}$$

### Immunofluorescence

Immunofluorescence was performed to visualize Aβ pathology and localize the administered antibody in sagittal cryosections of brain tissue from mice that previously underwent PET imaging as well as from 18 to 20 month old Tg-ArcSwe (*n* = 2) and WT (*n* = 2) mice that did not receive any antibody injection. Tissue sections (20 μm thick) were initially fixed in ice-cold methanol for 10 min and subsequently rinsed twice in phosphate-buffered saline (PBS) for 5 min each. To block unspecific binding and permeabilize the tissue, sections were incubated for 2 h in a blocking solution containing 5% normal goat serum (NGS) in PBS. After blocking, sections were washed three times for 5 min in PBS. Fibrin staining was performed by incubating the sections with 1101 diluted 1:1000 in PBS 0.05% Tween-20 overnight. Sections were washed three times for 5 min in PBS followed by incubation of goat anti-mouse Alexa Fluor 647-conjugated antibody (A21235, Invitrogen) diluted 1:200 in PBS 0.05% Tween-20 for 1 h at room temperature. Following this incubation, slides were washed twice for 5 min in PBS. To label Aβ fibrils, sections were then incubated with the luminescent conjugated oligothiophene (LCO) HS-84, diluted to a final concentration of 50 nM in PBS 0.05% Tween-20, for 15 min at room temperature under gentle shaking. Excess dye was removed by washing the slides three times for 5 min in PBS. Sections were mounted using a mounting medium containing DAPI and imaged using a Zeiss Observer Z.1 microscope with ZEN 3.7 software (Carl Zeiss Microimaging GmbH, Jena, Germany).

### PET imaging

Tg-ArcSwe (21 months; *n* = 3 per antibody) and WT (19–21 months; *n* = 2 per antibody) mice were administered 5.2 ± 0.73 kBq/g and 6.8 ± 0.84 kBq/g body weight of [^124^I]1101 and [^124^I]1101-scFv8D3, respectively. At 72 h post-injection, a 120 min PET scan (Mediso NanoPET/MR, Mediso Medical Imaging System, Hungary) was acquired, followed by a 5-minute CT scan (Mediso NanoSPECT/CT, Mediso Medical Imaging System). Anaesthesia was maintained throughout the scanning procedure using 3.5–4.0% sevoflurane in a 0.5 L/min flow of 50% oxygen and 50% medical air. PET data were reconstructed on a 160 × 160 × 128 grid with voxel dimensions of 0.5 × 0.5 × 0.6 mm³ using three-dimensional ordered-subsets expectation maximization (20 iterations). CT data were reconstructed using filtered back-projection. Processing of the PET and CT images was performed with Amide, version 1.0.4 (Loening and Gambhir 2003). The CT scans were manually aligned with a T2-weighted, MRI-based mouse brain atlas (Ma et al. 2005). The PET images were subsequently aligned with the CT image, and a region of interest representing the cerebrum (brain) was outlined.

After the CT scan, a terminal blood sample was obtained from the heart before the mice were perfused, and the brain and peripheral organs were collected using the same procedure as described above. Radioactivity in the tissues was quantified via γ-counting, and radioactivity concentrations were converted to SUV. Sagittal brain sections (20 μm) were prepared from the right hemispheres and exposed to a phosphor imaging plate (MS, MultiSensitive, PerkinElmer) for 10 days. Plates were scanned in a Typhoon phosphor imager (Cytiva).

### Statistical analyses

Statistical analyses were performed in GraphPad Prism 10.2.2 (GraphPad Software, Inc., San Diego, CA). The results are reported as mean ± standard deviation and statistical assessment was conducted by unpaired t-test or a two-way ANOVA with multiple comparisons test.

## Electronic supplementary material

Below is the link to the electronic supplementary material.

Supplementary Material 1

## Data Availability

The datasets used and/or analysed during the current study are available from the corresponding author on reasonable request.

## Abbreviations

AD: Alzheimer’s disease
Aβ: Amyloid beta
BBB: Blood-brain barrier
Cau: Caudate
Cbl: Cerebellum
CDR: Complementarity-determining region
CT: Computed tomography
Ctx: Cortex
ELISA: Enzyme-linked immunosorbent assay
Fab: Fragment antigen-binding
FBP: Fibrin-binding peptide
Hipp: Hippocampus
IgG: Immunoglobulin G
LCO: Luminescent conjugated oligothiophene
mTfR: Mouse transferrin receptor
NHS: N-hydroxysuccinimide
PET: Positron emission tomography
ROI: Region of interest
scFv: Single-chain variable fragment
SUV: Standardized uptake value
TfR: Transferrin receptor
Thl: Thalamus
Wb: Whole brain
WT: Wild-type
¹²⁴I: Iodine-124
¹²⁵I: Iodine-125
