# Supplementary material for: Brain-penetrating peptide and antibody radioligands for proof-of-concept PET imaging of fibrin in Alzheimer’s disease
Source: EJNMMI Radiopharm Chem. 2025 Sep 8;10:59. doi: 10.1186/s41181-025-00383-2 (PMC12417348; doi:10.1186/s41181-025-00383-2)
Supplement: Supplementary file 1 — Supplementary Material 1 [file 41181_2025_383_MOESM1_ESM.docx]

**Supplementary information**

**Figure S1**. Structure of FBP.

**
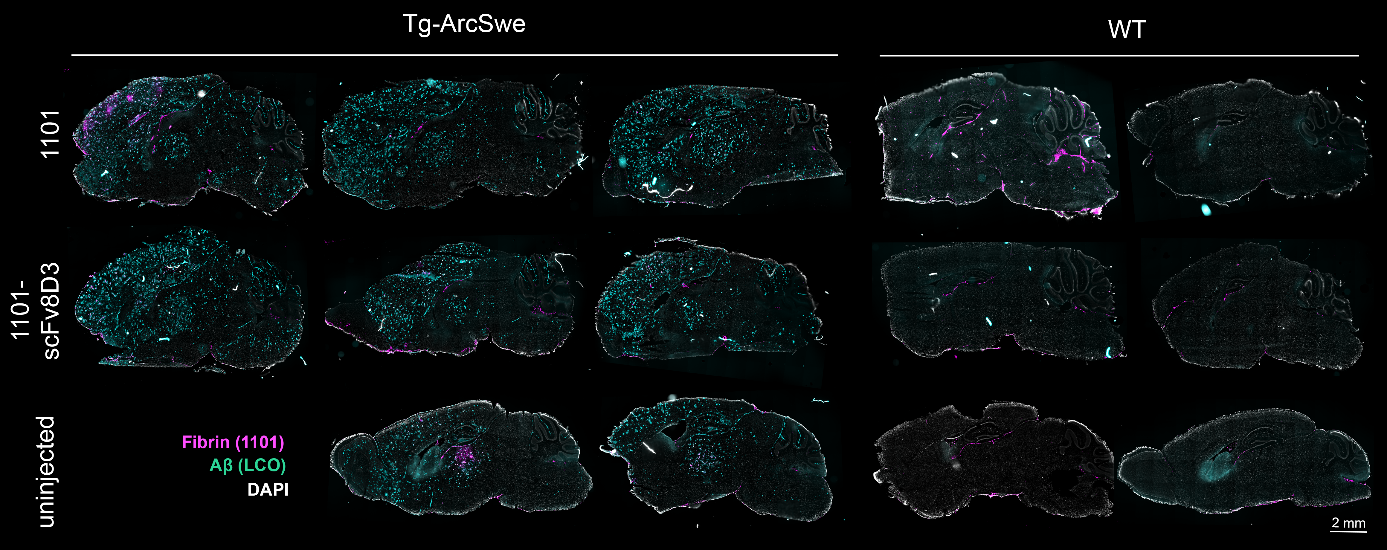
**

**Figure S2.** Fluorescent staining of fibrin (magenta; antibody 1101) and Aβ (cyan; LCO HS-84) in brain tissue from PET scanned Tg-ArcSwe and WT mice injected with [^124^I]1101 or [^124^I]1101-scFv8D3 and from uninjected mice of both genotypes.

**Figure S3**. **ImmunoPET with [^124^I]1101 and [^124^I]1101-scFv8D3.** Sagittal PET and autoradiography images of all Tg-ArcSwe and WT mice included in the study, acquired 72 h post injection of **A**) [^124^I]1101 or **B**) [^124^I]1101-scFv8D3, expressed as standardized uptake value (SUV, upper panel) or brain to blood ratio (middle panel). Note the different scales for the two antibody ligands. Ex vivo autoradiography (lower panel) from saline perfused, PET scanned mice.


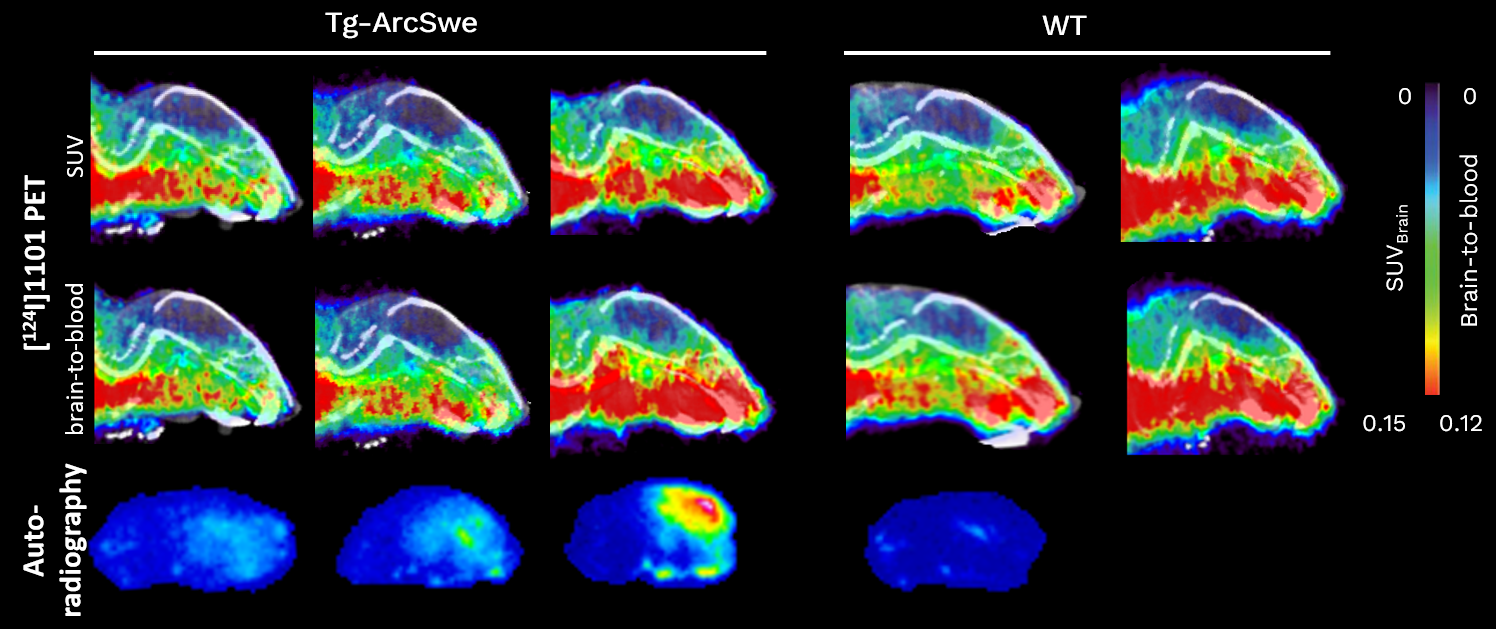

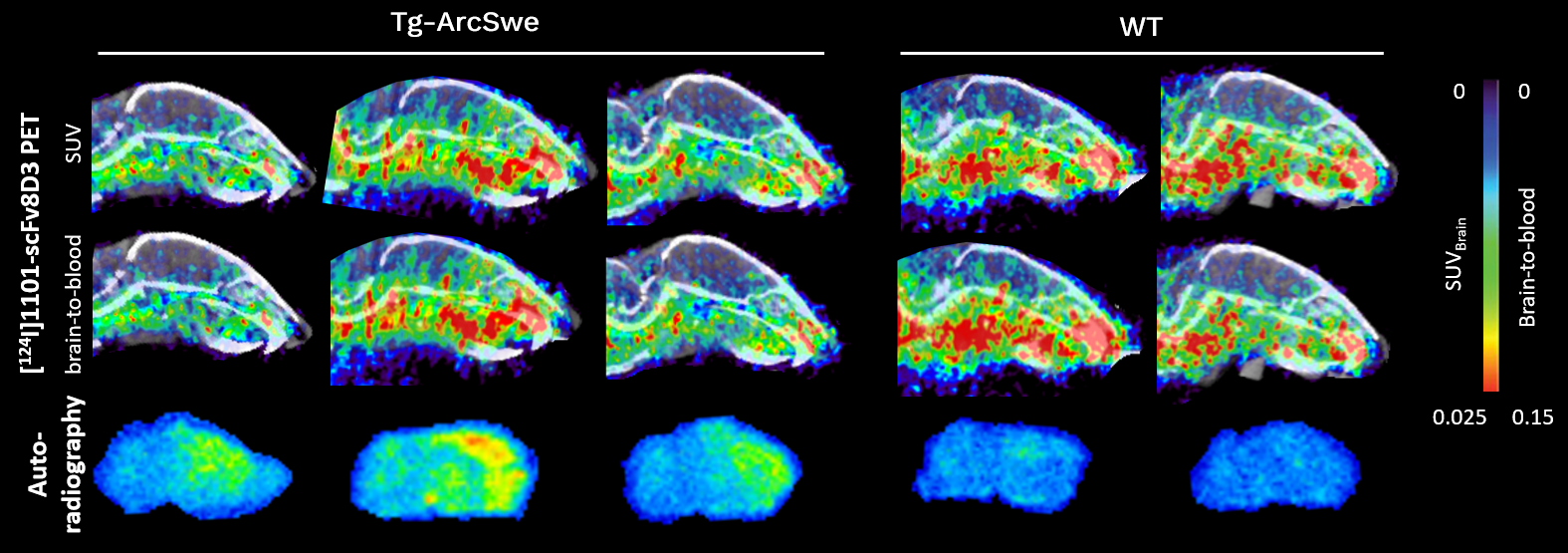


**A)**

**B)**
